# Demyelination and remyelination detected in an alternative cuprizone mouse model of multiple sclerosis with 7.0 T multiparameter magnetic resonance imaging

**DOI:** 10.1038/s41598-021-90597-6

**Published:** 2021-05-26

**Authors:** Shuang Ding, Yu Guo, Xiaoya Chen, Silin Du, Yongliang Han, Zichun Yan, Qiyuan Zhu, Yongmei Li

**Affiliations:** 1 Department of Radiology, The First Affiliated Hospital of Chongqing Medical University, Chongqing, 400016 China; 2 Department of Radiology, Daping Hospital, Army Medical University, Chongqing, 400016 China

**Keywords:** Multiple sclerosis, Biotechnology

## Abstract

The aim of this study was to investigate the mechanisms underlying demyelination and remyelination with 7.0 T multiparameter magnetic resonance imaging (MRI) in an alternative cuprizone (CPZ) mouse model of multiple sclerosis (MS). Sixty mice were divided into six groups (n = 10, each), and these groups were imaged with 7.0 T multiparameter MRI and treated with an alternative CPZ administration schedule. T_2_-weighted imaging (T_2_WI), susceptibility-weighted imaging (SWI), and diffusion tensor imaging (DTI) were used to compare the splenium of the corpus callosum (sCC) among the groups. Prussian blue and Luxol fast blue staining were performed to assess pathology. The correlations of the mean grayscale value (mGSV) of the pathology results and the MRI metrics were analyzed to evaluate the multiparameter MRI results. One-way ANOVA and post hoc comparison showed that the normalized T_2_WI (T_2_-nor), fractional anisotropy (FA), mean diffusivity (MD), radial diffusivity (RD), and axial diffusivity (AD) values were significantly different among the six groups, while the mean phase (Φ) value of SWI was not significantly different among the groups. Correlation analysis showed that the correlation between the T_2_-nor and mGSV was higher than that among the other values. The correlations among the FA, RD, MD, and mGSV remained instructive. In conclusion, ultrahigh-field multiparameter MRI can reflect the pathological changes associated with and the underlying mechanisms of demyelination and remyelination in MS after the successful establishment of an acute CPZ-induced model.

## Introduction

Multiple sclerosis (MS) is the most common myelin degeneration disease of the central nervous system^1^. The principal pathological mechanisms underlying MS include myelin sheaths loss, inflammation, edema, and abnormal iron deposition, among others^2,3^. Previous studies have indicated that spontaneous remyelination occurs after demyelination in some MS patients^4^. However, it is difficult to define the extent of changes in myelin during demyelination and remyelination due to the complex pathological mechanisms underlying MS.

Currently, multiparameter magnetic resonance imaging (MRI), which can detect different pathological mechanisms, is the best noninvasive method for the in vivo detection of MS^2,5^. An example of multiparameter MRI is conventional T_2_-weighted imaging (T_2_WI), which is mainly affected by tissue water composition and can sensitivity detect inflammation and edema in tissues^4,6^. Moreover, in regions with dense myelin, such as the corpus callosum (CC), the signal intensity of T_2_WI is inversely proportional to the myelin content^7^. Susceptibility-weighted imaging (SWI) mainly reflects the magnetic susceptibility of tissues in a magnetic field^8,9^. The variation in magnetic susceptibility reflected in phase images of SWI may be due to two factors: the increase in paramagnetism caused by abnormal iron deposition in the lesion or the decrease in diamagnetism caused by myelin loss^10,11^. Diffusion tensor imaging (DTI) can detect changes in both the microscopic and structural integrity of axons and the myelin sheaths in DTI metrics by detecting the diffusion of water molecules and generating diffusion tensor metric maps^12,13^. The fractional anisotropy (FA) value indicates microstructural integrity, the mean diffusivity (MD) value indicates water molecule diffusivity in tissues, the radial diffusivity (RD) value indicates the status of axonal fibers considering myelin sheaths diameter and density, and the axial diffusivity (AD) value indicates axial damage and is mainly used for axons^14^. Therefore, the changes in these MR metrics are of great significance for revealing the pathological mechanisms underlying the demyelination and remyelination in MS.

The cuprizone (CPZ)-induced mouse model is often used to study demyelination and remyelination due to its unique pathological mechanisms, and CPZ is essential to simulate the key activities of myelin-forming oligodendrocytes (OLs) in demyelination and remyelination^15–17^. The caudal segment of the CC, especially the central part of the splenium of the corpus callosum (sCC), is the most focused region and is known to be particularly sensitive to treatment with toxic compounds. In this model, the apoptosis of OLs leads to demyelination, which is accompanied by typical pathological manifestations. After withdrawing CPZ to allow remyelination, oligodendrocyte precursor cells travel to the myelin-deficient area, differentiate into mature OLs, and then rewrap axons to achieve complete remyelination^16^. The traditional CPZ model was improved by the quantitative oral gavage method in the study of Zhen et al.^18^. These authors proposed that the alternative CPZ model could effectively reduce the variability in the process of demyelination in individual animals^18^. Therefore, the results of comparisons among groups could be more reliable. However, the alternative model has not been applied in the field of MRI.

Based on previous research, we established an alternative CPZ mouse model that was subjected to 7.0 T multiparameter MRI with T_2_WI, SWI, and DTI sequences and pathological analysis at a series of dynamic time points to comprehensively explore the mechanisms underlying demyelination and remyelination and provide further MRI evidence for the clinical detection of MS.

## Methods

### Animal model

The current experiments were performed in strict accordance with the guidelines and regulations of the Laboratory Animal Guideline for Ethical Review of Animal Welfare for China (No. GB/T 35892-2018). All the procedures performed in the studies involving animals were supervised and approved by the ethical standards of the Animal Ethics Committee of Chongqing Medical University (Research Ethics No. 2019-065). All the animal experiments followed the ethical guidelines of ARRIVE.

Six-week-old male C57BL/6J specific pathogen-free mice were provided by the Experimental Animal Center of Chongqing Medical University, housed in individually ventilated cages, placed in an environment with alternating light/dark cycles of 12 h and a room temperature of 24 °C, and provided with clean drinking water and sufficient food. All the mice were administered 0.5% sodium carboxymethyl cellulose (CMCNa; Solarbio, China) by gavage once a day for one week to adapt to irritation.

Thereafter, the mice were divided into a 3-week demyelination group (3w group, n = 10), a 4-week demyelination group (4w group, n = 10), a 6-week demyelination group (6w group, n = 10), a 6-week demyelination followed by 4-week remyelination group (10w group, n = 10), a 6-week demyelination followed seven-week remyelination group (13w group, n = 10), and a group without CPZ administration that served as the healthy control group (0w group, n = 10).

**Figure 1:**
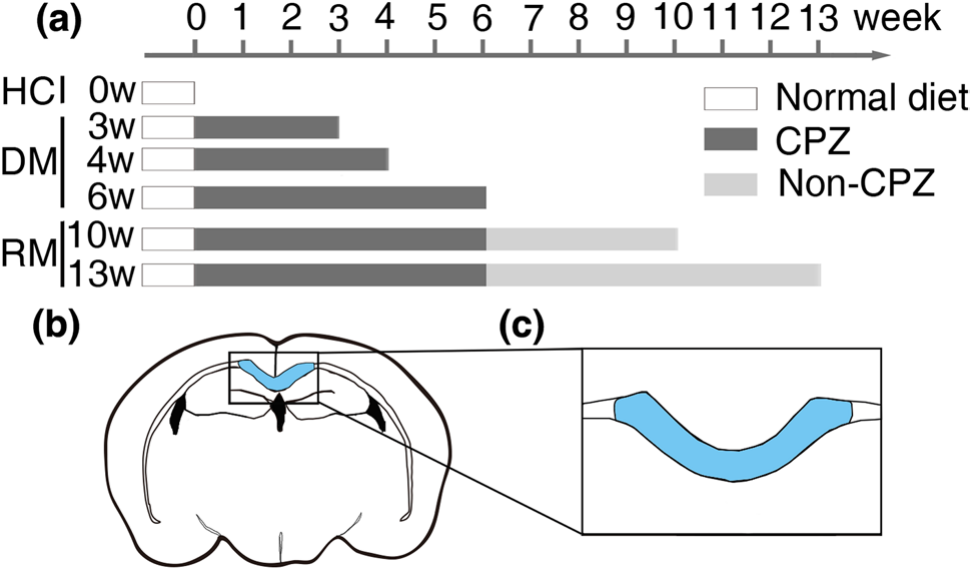
Experimental design and ROI selection. (**a**) According to the schedule of the CPZ administration, MRI and pathological examinations were performed after each group arrived at the time point, respectively; (**b**) The sCC was selected as the ROI (the blue area) to calculate metrics of MRI; (**c**) The sCC was selected as the ROI (the blue area) to calculate the mGSV of all sections of each mouse.

The CPZ model was established by the daily oral gavage of a 10 ml/kg CPZ-CMCNa suspension to induce the demyelination process. The suspension was freshly prepared using 400 mg/kg CPZ powder (Sigma-Aldrich, America) mixed with 0.5% CMCNa. At the end of six weeks of gavage, CPZ was no longer administered to the mice in the 10w group and 13w group, and these mice were fed normal chow to induce the process of spontaneous remyelination. MRI scans and pathology examinations were performed after each group reached at the indicated time point, as shown in Fig. 1a.

### MRI acquisition

All the MRI scans were acquired with Bruker 7.0 T small animal magnetic resonance (Bruker BioSpec USR 70/20, Paravision 6.0.1 operating system; Bruker, Germany). The mice were placed in the head prone position and continuously given inhaled isoflurane gas (induction concentration 4%, continuous concentration 1.5%) for anesthesia. The vital signs of the mice were monitored via a supporting physiological monitor.

The MRI sequences and parameters used in the experiment are listed as follows: ① T_2_WI was acquired with a 2D rapid acquisition with refocused echoes (RARE) sequence, RARE factor = 6, repetition time (TR) = 3000 ms, echo time (TE) = 45 ms, field-of-view (FOV) = 25 mm × 25 mm, matrix = 256 × 256, slice thickness = 0.6 mm, number of slices = 22, scan time = 8 min 24 s; ② SWI was obtained from a 2D fast low angle shot sequence, TR = 767 ms, TE = 17 ms, FOV = 25 mm × 25 mm, matrix = 384 × 384, slice thickness = 0.6 mm, number of slices = 22, scan time = 20 min 28 s; and ③ DTI was acquired with a diffusion weighted echo planar imaging sequence, TR = 3000 ms, TE = 40 ms, FOV = 20 mm × 20 mm, matrix = 128 × 128, slice thickness = 1.0 mm, number of slices = 22, b value = 0 and 800 s/mm^2^, 30 directions, scan time = 24 min 48 s. All the mice were subjected to the same scan sequences and parameters.

### MRI analysis

The MRI data were analyzed with the region of interest (ROI)-based method. According to the online atlas (http://mouse.brain-map.org/static/atlas/), the splenium of the corpus callosum (sCC) was selected as the ROI at the same place in all the mice. Two physicians with more than 5 years of experience in MRI diagnosis manually delineated the ROI independently, and the measurements of the given ROI were averaged over the two adjacent slices (Fig. 1b). After passing the intraclass correlation coefficient of ROI volume (Supplementary Table S1), the final value was averaged by the two calculation results.

The T_2_WI data were processed in a normalized-based method. In brief, the ROIs of the sCC (T_2_-sCC) and the third ventricle (T_2_-V3) were delineated manually at the same slice in ITK-SNAP (http://www.itksnap.org/pmwiki/pmwiki.php; ITK-SNAP, version 3.8.0). Then, the two corresponding ROIs were imported into MATLAB (MATLAB, version 2013b 8.2.0.701; MathWorks, America), and the normalized T_2_WI (T_2_-nor) value was calculated by Formula (1) in MATLAB.

1 $$ {\text{T}}_{2} - {\text{nor}} = {\text{T}}_{2} - {\text{sCC}}/{\text{T}}_{2} - {\text{V}}3. $$

The SWI measurements were performed on the phase image according to Formula (2). In brief, phase images were reconstructed using a 64 × 64 Hanning Filter of SPIN software (http://www.mrimaging.com/; SPIN SVN, revision 1591) to calculate the phase unwrapping and remove the low-frequency components. In addition, the ROI was delineated in the filtered phase image to extract the mean phase (Φ) value of SWI after adjusting the window width and level.

2 $$ {\text{F}} = - \gamma \cdot{\text{c}}\cdot{\text{V}}\cdot\Delta {\text{x}}\cdot{\text{B}}0\cdot{\text{TE}}. $$

Φ is the phase of SWI; c is the iron content; V is the voxel; Δx is the changes in molar magnetic susceptibility between tissues.

The DTI data were postprocessed using FSL Software (https://fsl.fmrib.ox.ac.uk/; FMRIB Software Library, version 5.0.9). First, images were reconstructed to generate the FA map, λ_1_ map, λ_2_ map, λ_3_ map, and other metric maps. The ROI was manually delineated by ITK-SNAP on the b0 image, and metric maps were calculated into the MD value, RD value, and AD value by Formulas (3), (4), and (5) in FSL, respectively.

3 $$ {\text{MD}} = ({\text{l}}_{1} + {\text{l}}_{2} + {\text{l}}_{3} )/3, $$

4 $$ {\text{RD}} = ({\text{l}}_{2} + {\text{l}}_{3} )/2, $$

5 $$ {\text{AD}} = {\text{l}}_{1} . $$

### Pathological staining

The mice were still under deep anesthesia after the scan. The mice immediately underwent surgery to puncture the left atrium with an infusion needle after thoracotomy, and then, the mice were injected with 150 ml phosphate-buffered saline to lavage the systemic blood. Then, 150 ml 4% paraformaldehyde solution was added to fix the specimen. Finally, the head was removed, and the brain tissues were extracted and subsequently stored in 4% paraformaldehyde solution for 48 h. After being embedded in paraffin, serial sections in the coronal position with a thickness of 5 μm starting − 1.5 mm from bregma were cut for staining procedures as follows.

Prussian blue (PB) staining was carried out to measure nonheme iron deposition. In brief, all the sections were deparaffinized at 60 °C for three hours and hydrated in a series of xylene and gradient ethanol as preprocessed. Then, parts of the sections were incubated in equal parts of 20% aqueous hydrochloric acid and 10% potassium ferrocyanide for 30 min. The excess dye was washed away, and the samples were counterstained in Nuclear Fast Red solution for 3 min (all reagents were from the PB staining kit; Sigma-Aldrich, America). To further confirm the reliability of PB staining, a group of positive control mice (n = 5) was intraperitoneally injected with iron-dextran (1.0 g/kg, once a week for 6 weeks), and pathological brain tissues were extracted and stained with PB using the above methods.

Luxol Fast Blue (LFB) staining was used to identify the myelin sheaths. The rest of the sections were incubated for 5 h in LFB solution that was pre-heated for eight hours (Sigma-Aldrich, America). Then, the excess dye was washed away, and the samples were differentiated alternately in 0.5% lithium carbonate solution and 75% ethanol solution and counterstained in eosin solution for 5 min. Finally, those sections were redehydrated using gradient ethanol, cleared in xylene, and then mounted with neutral resin. The sections were photographed with the same parameters by a Carl Zeiss Axioscope5 optical microscope (Carl Zeiss, Germany).

### Pathological analysis

Serial sections for PB staining (10 sections) and LFB staining (10 sections) from each mouse were analyzed at 200 × magnification by ImageJ software (https://imagej.nih.gov/ij/; ImageJ, with Java version 1.8.0.172).

The semiquantitative scoring system of PB staining was used to evaluate iron deposition in sCC (Fig. 1c). The scoring criteria were as follows: 0 = no iron deposition; 1 = iron deposition area (S_PB_) less than 5% of the FOV; 2 = S_PB_ accounts for 5% ~ 25% of the FOV; 3 = S_PB_ accounts for 25% ~ 50% of the FOV; 4 = S_PB_ accounts for 50% ~ 75% of the FOV; and 5 = S_PB_ accounts for more than 75% of the FOV.

The mean grayscale value (mGSV; range 0–255) of LFB staining was measured as a semiquantitative metric of the myelin sheaths in ImageJ software. In brief, the LFB-stained sections were transformed into 8-bit grayscale images, and then, the sCC was manually delineated on all slices over the same region of each mouse. The mGSV was calculated and corrected in the selected region by built-in algorithms (Fig. 1c).

### Statistics

Two mice in the 10w group that died during administration were excluded; hence, a total of 58 mice were included in the statistical analysis. The data analysis was performed with SPSS statistical software (IBM SPSS, version 24; America). The results are expressed as the mean ± standard deviation ($\overline{x } \pm SD$). *One-way ANOVA* was performed for data that met the homogeneity of variance, and post hoc comparisons were corrected by the *Bonferroni* method. Otherwise, the *Brown Forsythe* method and the *Dunnett correction* were used. *Spearman's rank correlation* was used to analyze pairwise correlations between multiparameter MR metrics and mGSV. *p < 0.05, **p < 0.01, and ***p < 0.001 were considered significantly different.

## Results

### Multiparameter MRI findings

**Figure 2:**
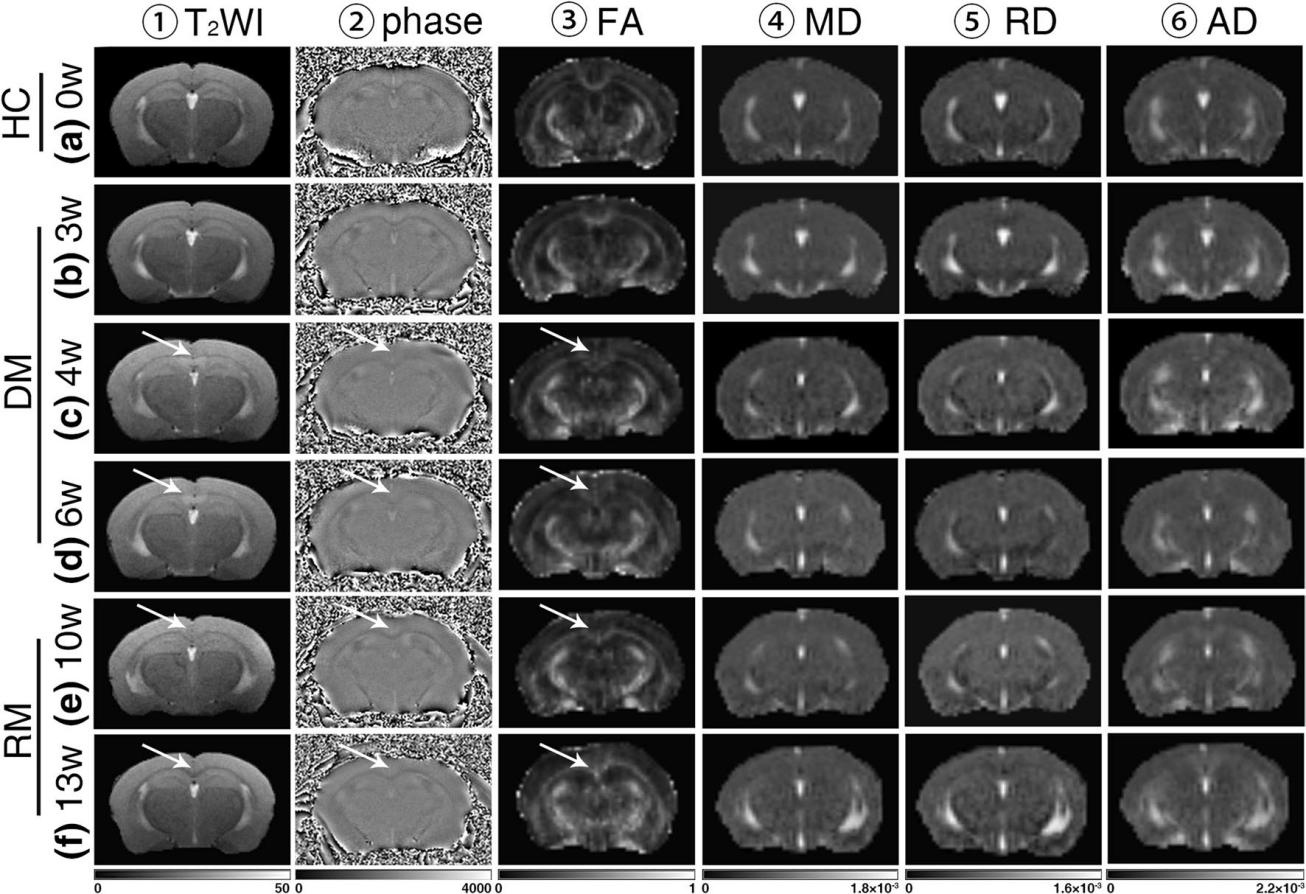
multiparameter MRI findings. The white arrow indicates the abnormal signal of the sCC lesion. Columns represent groups of CPZ administration, (**a–f**) groups of models by chronological order: (**a**) control tissues in the 0w group; (**b**), ormal-appearing imaging in the 3w group; (**c**) the abnormal signal of sCC in multiparameter MRI in the 4w group; (**d**) those abnormal signals persisted and aggravated in the 6w group; (**e**) a lesion which underwent the remyelination process in the 10w group; (**f**) abnormal signals were recovered for the most part at the 13w group. And rows represent multiparameter MRI sequences: (①) ~ (⑥), sequences of multiparameter MRI: (①), T_2_WI; (②), phase image; (③), FA map; (④), MD map; (⑤), RD map; (⑥), AD map.

None of the obvious abnormal signal changes were observed in the 0w group (Fig. 2a① ~ a⑥). During the demyelination process, no obvious abnormal signals were found in the sCC in the T_2_WI, phase images of the 3w group or in the DTI metric maps (Fig. 2b① ~ b⑥). However, persistent and aggravated abnormal signals of the sCC in multiparameter MRI, including crescent-shaped hyperintensity in T_2_WI and hyperintensity in phase imaging, and damaged structures, particularly in the FA map of DTI, were observed in the 4w group (Fig. 2c① ~ c⑥) and 6w group (Fig. 2d① ~ d⑥). During the remyelination process, these abnormal signals of the sCC lesions were observed to gradually decrease in the 10w group (Fig. 2e① ~ e⑥), eventually returning to healthy control levels in the 13w group (Fig. 2f① ~ f⑥).

### Variations in multiparameter MRI metrics among groups in the CPZ mouse model

**Figure 3:**
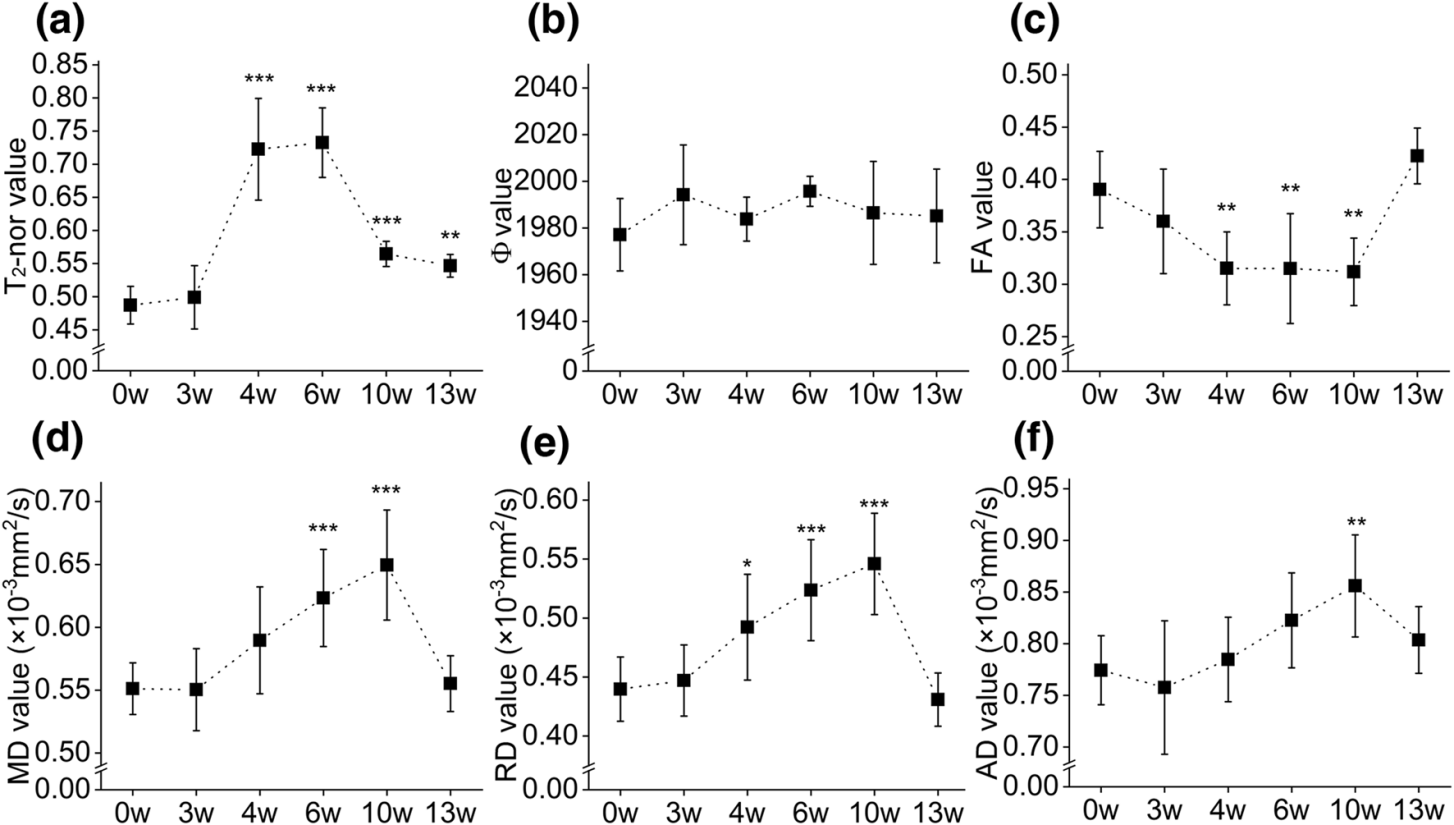
Trends and comparisons of MRI metrics of time points of mice model. (**a**) the T_2_-nor value was increased during the demyelination process and decreased during the remyelination process; (**b**) the Φ value of SWI has remained slight fluctuated during the whole process; (**c**) FA value was decreased during the demyelination process, reached the valley at the 10w group, then increased in the 13w group; (**d**) MD value was decreased slightly to the valley at 3w group then increased continuously till reached the peak at the 10w group, finally following decreased at 13w group; (**e**) RD value was increased till reached the peak at the 10w group, then following decreased at 13w group; (**f**) the trend polyline of the AD value owned the valley at the 3w group and the peak at the 10w group. Compared with the 0w group, statistically differences are *p < 0.05, **p < 0.01, ***p < 0.001.

*One-way ANOVA* showed a significant difference in the T_2_-nor values among the six groups (*F* = 28.570, *p* < 0.001; Table 1). Post hoc comparisons showed significant differences between the 0w group and 4w group, 0w group and 6w group, 0w group and 10w group, and 0w group and 13w group (all *p* < 0.01; Table 2, Fig. 3a). However, no significant difference in the Φ value was found among those groups by *one-way ANOVA* (*F* = 1.666, *p* = 0.168; Table 1, Fig. 3b). There were significant differences among the groups in all four diffusion tensor metrics of DTI (all *p* < 0.001; Table 1). Post hoc comparison showed significant differences between the 0w group and 4w group, 0w group and 6w group, and 0w group and 10w group in FA (all *p* < 0.01; Table 2, Fig. 3c). Moreover, significant differences between the 0w group and 6w group, 0w group and 10w group in MD were observed (all *p* < 0.001; Table 2, Fig. 3d). Similar to the FA, there were significant differences between the 0w group and the 4w group, 6w group, and 10w group, respectively (all *p* < 0.05; Table 2, Fig. 3e). However, after the post hoc comparison in AD, a significant difference was noted only between the 0w group and 10w group (*p* = 0.006; Table 2, Fig. 3f). Supplementary Table S2 provides more supplemental information about the pairwise multiple post hoc comparisons for MR metrics.

**Table 1:** One-way ANOVA of MRI metrics in the splenium of the corpus callosum of each group.

Groups of mice model		n	T_2_-nor value		Φ value	FA value	MD value (× 10^-3^mm^2^/s)	RD value (× 10^-3^mm^2^/s)	AD value (× 10^-3^mm^2^/s)
0w	10			0.487 ± 0.028	1977.092 ± 15.512	0.390 ± 0.037	0.551 ± 0.021	0.440 ± 0.027	0.774 ± 0.033
3w	10			0.499 ± 0.048	1994.208 ± 21.371	0.360 ± 0.050	0.550 ± 0.033	0.447 ± 0.030	0.758 ± 0.065
4w	10			0.722 ± 0.077	1983.789 ± 9.448	0.315 ± 0.035	0.590 ± 0.042	0.492 ± 0.045	0.785 ± 0.040
6w	10			0.733 ± 0.052	1995.690 ± 6.412	0.314 ± 0.053	0.623 ± 0.039	0.524 ± 0.043	0.823 ± 0.046
10w	8			0.565 ± 0.019	1986.446 ± 22.071	0.311 ± 0.032	0.650 ± 0.044	0.546 ± 0.043	0.856 ± 0.049
13w	10			0.547 ± 0.017	1985.123 ± 20.083	0.423 ± 0.027	0.555 ± 0.022	0.430 ± 0.055	0.804 ± 0.032
*F*, *p*	–			57.520, < 0.001***	1.666, 0.168	13.050, < 0.001***	13.767, < 0.001***	16.489, < 0.001***	5.481, < 0.001***

Normally distributed data are represented by $\overline{x } \pm SD$. Statistically differences are *p < 0.05, **p < 0.01, ***p < 0.001.

**Table 2:** Post hoc comparison of MRI metrics in the splenium of the corpus callosum of each group.

Post hoc comparison (*t*, *p*)					
	T_2_-nor	FA	MD	RD	AD
0w vs 3w	− 0.682, 1.000	1.692, 1.000	0.049, 1.000	− 0.455, 1.000	0.818, 1.000
0w vs 4w	− 9.092, < 0.001***	4.192, 0.002**	− 2.515, 0.226	− 3.272, 0.029*	− 0.509, 1.000
0w vs 6w	− 13.009, < 0.001***	4.203, 0.002**	− 4.719, < 0.001***	− 5.227, < 0.001***	− 2.369, 0.324
0w vs 10w	− 6.926, < 0.001***	4.130, 0.002**	− 6.065, < 0.001***	− 6.238, < 0.001***	− 3.778, 0.006**
0w vs 13w	− 5.692, 0.001**	− 1.788, 1.000	− 0.265, 1.000	0.552, 1.000	− 1.437, 1.000

As there was no significant difference in the *one-way ANOVA*, the Φ value was not processed in the post hoc comparison. Statistically differences are *p < 0.05, **p < 0.01, ***p < 0.001.

### Pathological staining findings

**Figure 4:**
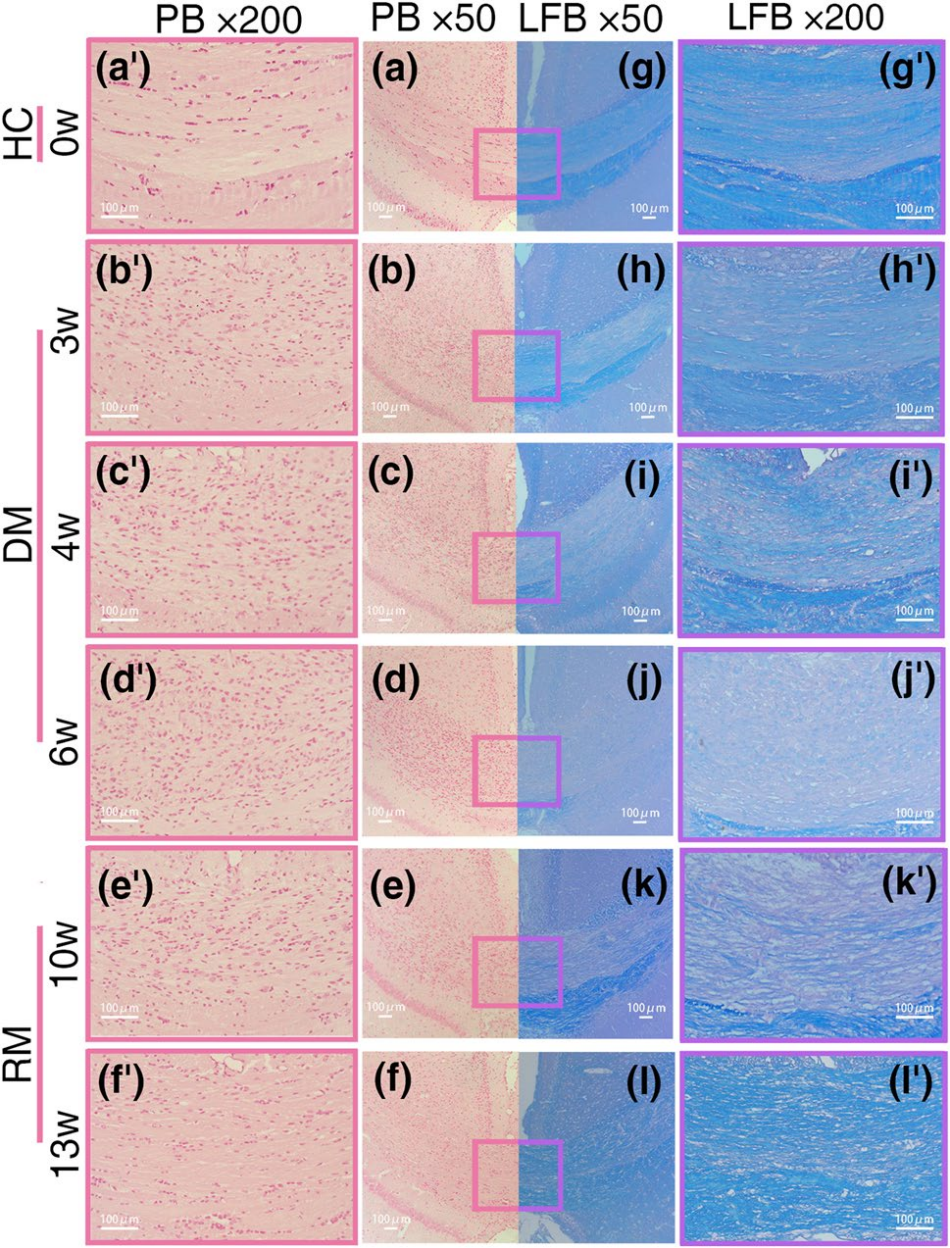
Pathology staining findings. PB staining could chemically react the non-blood hemosiderin to form ferrocyanide depositions but none of them were found in those sections, and all cell nuclei in the tissues were stained into brownish-red by NFS counterstaining. (**a**–**f**) a typical section with PB staining (× 50) of each group; (**a**′–**f**′) In situ magnification (× 200) of section. In the LFB staining, the myelin sheaths were stained with blue, and the cytoplasm of axons was counterstained with eosin to light red. (**g**–**l**) a typical section with LFB staining (× 50) of each group, (**g′**–**l′**) In situ magnification (× 200) of section. The selected sections with PB staining and LFB staining from the same mouse.

The PB staining of each group showed no blue ferrocyanide deposits in the sCC lesion, i.e., none of the indicators of abnormal iron deposition in the tissues were found (Fig. 4a–f,a′–f′). In addition, in the 0w group, the cell nuclei were regularly arranged (Fig. 4a′), while among the cells undergoing the demyelination process, the cell nucleus density increased along with the demyelination process (Fig. 4b′–d′). During remyelination, the nuclei gradually decreased in the 10w group (Fig. 4e′) compared with the 6w group and decreased in the 13w group (Fig. 4f′). In the positive control group, abnormal blue punctate deposits were observed in the cerebral tissues of the choroid plexus and the paraventricular brain tissues; however, no obvious iron deposits were found in the CC (Supplementary Fig. S1), which showed the reliability of PB staining in the current study.

In LFB staining, the myelin sheaths of the 0w group were arranged horizontally with uniform staining, and the cytoplasm was not exposed (Fig. 4g,g′), while the loss of myelin sheaths and the unveiling of the cytoplasm in the sCC lesion gradually increased with the increased progression of the demyelination process from the 3w group to the 6w group (Fig. 4h–j,h′–j′). During remyelination, the regenerating myelin sheaths gradually recovered compared to the demyelinated tissues in the 10w group (Fig. 4k,k′). In the final stage of the experiment in the 13w group, the myelin tissue had basically regenerated but was still not as healthy as that observed in normal tissue (Fig. 4l,l′).

### Comparison of the metrics of pathological staining among groups in the CPZ mouse model

The score for semiquantitative PB staining was 0 for each group (Table 3), which was consistent with the direct observation of PB staining.

**Table 3:** One-way ANOVA of semi-quantitative pathological staining in the splenium of the corpus callosum of each group.

Groups of mice model	Sample of included mice in a group (n)	Sections for each mouse	Score of PB	mGSV
0w	10	10	0	137.138 ± 5.010
3w	10	10	0	157.923 ± 8.051^§^
4w	10	10	0	168.061 ± 13.787^§^
6w	10	10	0	168.640 ± 10.251^§^
10w	8	10	0	147.350 ± 7.487^§^
13w	10	10	0	145.561 ± 4.515^§^
*F*, *p*	–	–	–	20.562, < 0.001

Normally distributed data are represented by $\overline{x } \pm SD$. ^§^*p* for a statistically significant difference compared with the 0w group (*p* < 0.05). Statistically differences are *p < 0.05, **p < 0.01, ***p < 0.001.

For the LFB staining, the content of the myelin sheaths in the control tissues had a lower gray value in the grayscale images, as the myelin sheaths were lost; in addition, the blue staining of the sCC decreased in the demyelination and remyelination groups, and then, it becomes closer to white color (for mGSV is 255) in the grayscale images. For the semiquantitative analysis of LFB staining among the six groups, *one-way ANOVA* showed a significant difference in the mGSV (Table 3). The post hoc comparisons showed that each group of the CPZ-induced model exhibited a significant difference compared with the 0w group Supplementary Table S3, Supplementary Fig. S2).

### Correlations of metrics between multiparameter MRI and pathological staining

**Figure 5:**
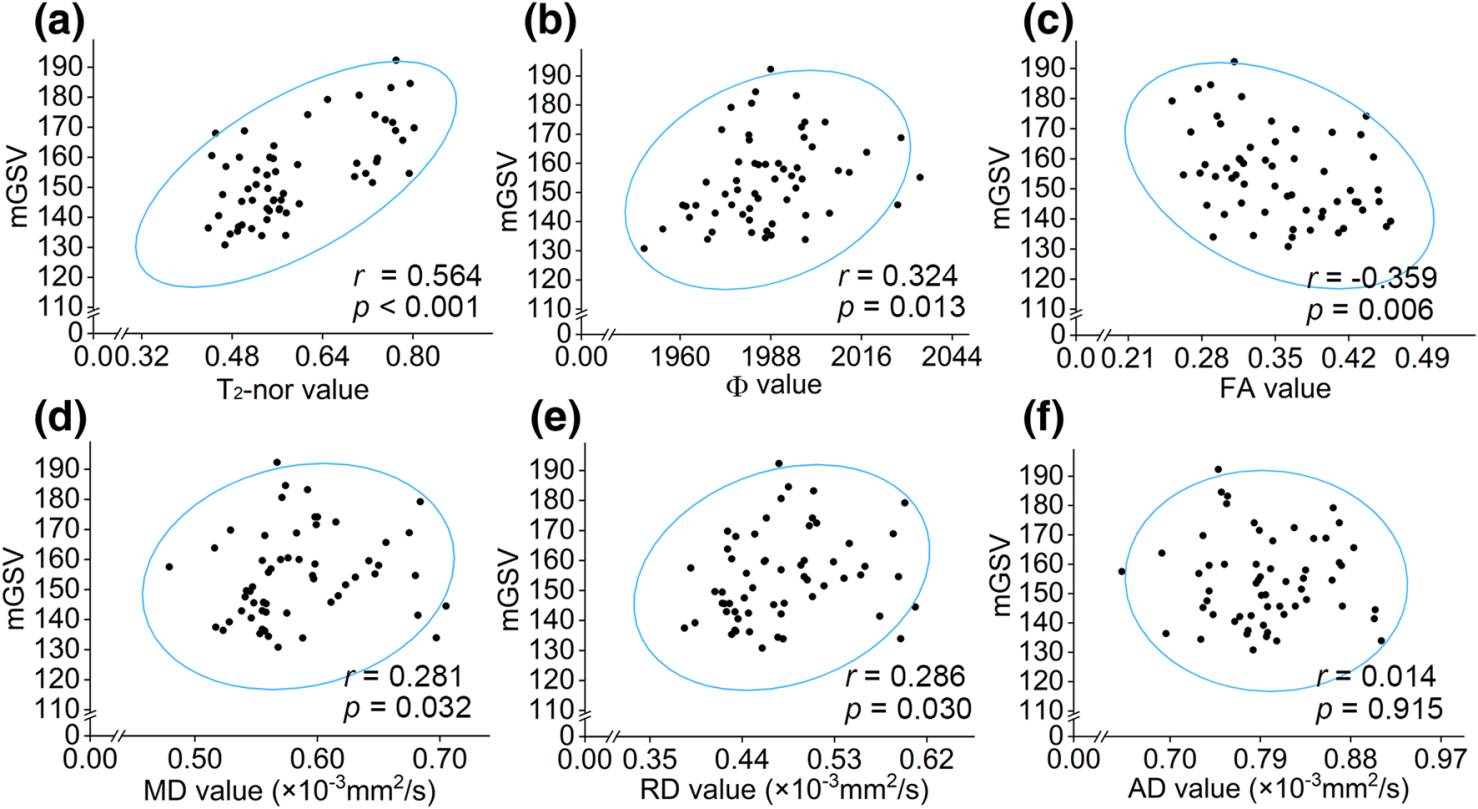
Correlations between multiparameter MRI metrics and semi-quantitative pathological staining. (**a**–**f**) *Spearman's rank correlation* analysis: (**a**) T_2_**-**nor value; (**b**) Φ value; (**c**) FA value; (**d**) MD value; (**e**) RD value; (**f**) AD value, respectively. The blue ellipse represents the confidence ellipsoid.

The results showed a significant and strong positive correlation between the T_2_-nor value and mGSV by *Spearman’s rank correlation* (*r* = 0.564, *p* < 0.001; Fig. 5a). There was also a significant, albeit lower, positive correlation between the Φ value and mGSV (*r* = 0.324, *p* = 0.013; Fig. 5b). Based on the DTI metrics, a significant negative correlation was found between the FA value and mGSV (*r* = − 0.359, *p* = 0.006; Fig. 5c), and there were lower positive correlations between the MD value and mGSV (*r* = 0.281, *p* = 0.032; Fig. 5d) and between the RD value and mGSV (*r* = 0.286, *p* = 0.030; Fig. 5e). However, no correlation between the AD value and mGSV was observed (*r* = 0.014, *p* = 0.915; Fig. 5f).

## Discussion

MS is one of the most common nontraumatic causes of disability in developed countries^1,19^. Spontaneous remyelination occurs after demyelination in MS, which is of profound significance to the prognosis of patients^5,19^. Therefore, to investigate the mechanisms underlying demyelination and remyelination, we established an alternative CPZ mouse model and detected the metrics of this model at a series of dynamic time points by ultrahigh-field multiparameter MRI.

A traditional CPZ model was established by ad libitum feeding of 0.2% (weight/weight) CPZ-containing fodder. For that reason, the model exhibited considerable individual differences and instability, which may have a great impact on the study’s results. Therefore, we established an alternative CPZ mouse model by oral gavage with the optimal dose of 400 mg/kg to induce demyelination and withdrawing CPZ to induce remyelination^18^. In the current study, a monosegmental lesion was found in the sCC by multiparameter MRI and pathology staining. This is similar to a previous study that investigated lesions in the middle and caudal slices of the CC in a 4-week CPZ-induced demyelination model by Wu et al.^20^. The reason for a single lesion may be due to the higher sensitivity of sCC to toxic induction than other tissues, indicating that CC can be affected in the short-term acute CPZ model.

Based on the successful establishment of the alternative CPZ model, we subsequently performed multiparameter MR scans for T_2_WI, SWI, and DTI to detect the three typical pathological manifestations. From the perspective of the MRI field, integrating the MRI and pathology results can help in comprehensively understanding the mechanisms of demyelination and remyelination in MS^2^.

Conventional T_2_WI can be used to quickly diagnose MS in clinical practice; however, most studies have focused on the morphological characteristics of the lesion observed with T_2_WI and rarely used any quantitative methods^5,21^. Therefore, we used cerebrospinal fluid as a calibration to normalize T_2_WI relaxation time to quantitatively measure the T_2_WI signal intensity^6,20^. Our results showed that during the demyelination process, the T_2_WI signal gradually changed to hyperintensity, which was accompanied by an increase in the T_2_-nor value. Except for the 3w group, the increase of T_2_-nor value in the 4w group and 6w group had significant differences than that of the 0w group, which may be due to the deterioration of demyelination caused by the aggravation of inducing time. Moreover, it may also suggest that the early changes of demyelination confirmed in pathology metric and staining may have not been sensitively detected in the 3w group by T_2_WI. This signal and metric changes from the 4w group to the 6w group may occur because in the demyelination process, edema has a stronger effect on the change in T_2_WI signal and T_2_-nor value, resulting in abnormal hyperintensity^22,23^. Then, during the remyelination process, the T_2_WI signal decreased to isointensity in the 10w group and then gradually returned to relative hypointensity in the 13w group, with a continuously decreased T_2_-nor value. This may indicate that as edema decreases, isointensity may contribute to the loss of myelin sheaths. As the signal returned to a relative hypointensity in the 13w group, but the T_2_-nor value consistent with mGSV value was not completely reduced to the level of the 0w group, which may indicate that myelin had not fully regenerated^24^. Moreover, there was a strong and significant correlation between T_2_-nor and mGSV. This result may indicate that T_2_-nor could reflect tissue edema and changes in myelin content during demyelination and remyelination^20,25^.

However, the T_2_WI signal is affected by many factors, not only related to inflammation and edema or the myelin sheaths. Therefore, additional methods with better specificity, such as myelin water fraction imaging (MWF) or ultrashort echo time (UTE)-magnetization transfer ratio (MTR)/FLAIR sequences^26–28^, could be used in subsequent studies for imaging myelin bilayer water molecules or myelin. However, these methods require more technical support, such as the MTR was limited with the spatial resolution and less reproducibility^29^, which is also one of the goals of our further research. However, the use of T_2_-weighted imaging suggests to a certain extent that T_2_-weighted imaging still has potential meaning for the relationship between tissue edema and even myelin^20,24,29^.

SWI has mainly been used in MS research to identify abnormal iron deposition and quantify the Φ value. However, most studies have not reported any changes in the magnetic sensitivity caused by myelin degeneration^10,30^. In the current study, no significant difference in the Φ value was observed among the groups, but a significant correlation between Φ and the mGSV was observed. Moreover, LFB staining results showed myelin loss in the lesion without iron deposition, as confirmed by PB staining.

These confusing SWI results may occur to the following reasons. From the perspective of iron deposition, previous studies have shown a decreasing trend of iron contained in the acute phase of MS^31^, which may lead to failure to detect iron deposition in the short-term acute model in this study. From the perspective of myelin loss, although signal changes in our phase images were noted, the quantification of the Φ value showed that SWI could not effectively distinguish changes in the myelin sheaths during demyelination and remyelination. This result indicates that SWI may not be a suitable choice for the detection of acute myelin loss without abnormal iron deposition. In recent years, quantitative susceptibility mapping (QSM) based on 3D multiecho flow compensated sequences have possibly had a better effect on the direct measurement of susceptibility^24,32^. However, due to the small volume of the mouse brain, the ears and other structures exert greater interference on the brain tissue, causing serious artifacts, and may require higher resolution and field strengths for research.

DTI has a wide range of applications for detecting MS due to its sensitivity to microstructural damage. The free diffusion of water molecules in brain tissue is restricted due to the cell membrane and myelin sheaths, especially for regions with dense and parallel white matter fibers (CCs). When myelin sheaths are lost or axons are damaged, the diffusion of water molecules can be sensitive to the effects^14^. In our results, there were significant differences among the groups in all four metrics. As these four metrics are not independent of the others, it is necessary to comprehensively evaluate the suggested pathological mechanisms in the context of variations in the values at dynamic time points^33^.

During the demyelination process, consistent with the T_2_WI, there is no significant statistical difference between the 3w group and the 0w group in all four diffusion tensor metrics, while it from the 4w group to the 6w group shows more significant changes. It also suggests the diffusion tensor metrics may not be able to detect early demyelination at the 3-week time point. After that, there was a continued increase from the 4w group to the 6w group compared with the 0w group in RD, which may be due to the deterioration of myelin loss and damage^14,25,34^. This possibility is also confirmed in LFB staining. While there was a biphasic change of AD, no significant difference was found at each time point of the entire demyelination process compared with the 0w group. Although there is no significant difference in AD, combined with the changes in RD, one possible explanation is that along with the progressive demyelination, the severe decreases in axonal packing density caused by loss of myelin sheaths and axons would leading an increase in extracellular water^35^, resulting in the larger continued increase of RD, and subsequent less significant increase of AD, besides, the relative elevation AD value in lesional fibers may also consistent with Wallerian degeneration^12,36^. For FA, the continuous increase in the RD may have a main and greater impact on FA, and such factors like reduction of myelin sheath integrity and fiber density, and edema may ultimately have led to the destruction of microscopic integrity of tissues^37,38^, which was manifested as a continuous decrease in FA from the 4w group to the 6w group^22^. For MD, in the 6w group, the progressive demyelination eventually led to cell membrane damage, the expansion of the tissue gap, an increase in the diffusion of free water molecules, and extracellular edema, etc.^13,39^, in other words, it is manifested as an increase in MD^33^.

During the remyelination process, FA continued to decrease in the 10w group, while MD, RD, and AD continued to increase in the 10w group, reaching extremums, besides, in the 10w group, there were significant differences of the four DTI metrics when compared with the 0w group. This result may indicate that as the factors inducing demyelination disappear, the damage continues until the level of repair is sufficient to overcome the damage^14,36^. It is worth noting that at the 10-week time point in the remyelination process, the four diffusion tensor metrics indicated the continual destruction of the sCC structure and the exacerbation of demyelination, while the T_2_-nor value was instead reduced. It may be due to there could be a large difference among the intracellular or extracellular water molecules balance, the cell membrane density, and the overall water content at this stage^39,40^. It may suggest that the two approaches are sensitive to different mechanisms where still has no definite conclusion^40^, and when the T_2_-nor value almost returned to its baseline value, DTI could still show alterations for a prolonged period. Finally, in the 13w group, the four diffusion tensor metrics almost recovered to the level of the 0w group, indicating that the integrity of the myelin sheaths and axons, the microscopic integrity, and membrane structure basically returned to 0w group level after the regeneration, combined with the T_2_-nor value and the mGSV value, it indicates that the remyelination is still incomplete^25^.

Combined with the correlation analysis with pathological staining, the metrics, except for AD, all had a significant correlation with the mGSV. Moreover, the FA value ranked higher than the RD value, and the MD value was the lowest. Furthermore, significant differences in both the FA value and RD value, but not in the MD value, between the 0w group and the 4w group were observed. It may be suggested to a certain extent that in the CPZ model, the comprehensive factors of axon and myelin sheaths damage reflect the decrease in the microscopic integrity and increase in the water molecule diffusion^41^. Radial myelin damage may contribute to this process to a large extent, while the effect of axons may be relatively weak or remain to be revealed^29,33,42^.

Several limitations of this study exist. First, the selected dynamic time points were asymmetrical, but there were typical representative characteristics at those selected time points in this study. Further studies are needed to continuously optimize the time point selection. Moreover, the scan sequences used in this study were based on the most typical pathological characteristics. It is still necessary to continue to optimize scan parameters and sequences in additional studies, for example, using higher field strengths and resolution or extending scan time; as well as above advanced sequences. Finally, the statistical power (Supplementary Table S4) for SWI is relatively low (60.3%), which may be because the sample size of the SWI sequence is still small. Therefore. the SWI results need to be further confirmed in future studies. It should also be pointed out that since no significant difference has been found between the experimental group and the healthy control group at some time points for MRI metrics, we could only speculate on the pathophysiological changes of those MRI metrics based on the existing trends and results, which may not inevitably fully explain those variations so that it still needs to be cautious for further confirmation.

In conclusion, based on the above comprehensive evaluation of these six sets of metrics, we speculate that multiparameter MRI detected with T_2_WI and DTI could sensitively reflect the demyelination and remyelination process in the alternative CPZ mouse model. Among those metrics, the T_2-nor_, FA, RD, and MD values showed sensitivity for detecting acute demyelination and remyelination, while the AD of DTI and SWI may not yet be considered. However, with the changes in different magnetic field strengths and models, the results need further verification.

## Supplementary Information

Supplementary Information.
