# Supplementary material for: Demyelination and remyelination detected in an alternative cuprizone mouse model of multiple sclerosis with 7.0 T multiparameter magnetic resonance imaging
Source: Sci Rep. 2021 May 26;11:11060. doi: 10.1038/s41598-021-90597-6 (PMC8155133; doi:10.1038/s41598-021-90597-6)
Supplement: Supplementary file 1 — Supplementary Information. [file 41598_2021_90597_MOESM1_ESM.pdf]

Demyelination and Remyelination Detected in an Alternative Cuprizone Mouse Model of Multiple Sclerosis with 7.0T Multiparameter Magnetic Resonance Imaging

Shuang Ding[1], Yu Guo[2], Xiaoya Chen[1], Silin Du[1], Yongliang Han[1], Zichun Yan[1], Qiyuan Zhu[1], Yongmei Li[1, *]

1 Department of Radiology, The First Affiliated Hospital of Chongqing Medical University, Chongqing 400016, China.

2 Department of Radiology, Daping Hospital, Army Medical University, Chongqing 400016, China.

*Corresponding Author: Yongmei Li. E-mail. Email address: lymzhang70@aliyun.com.

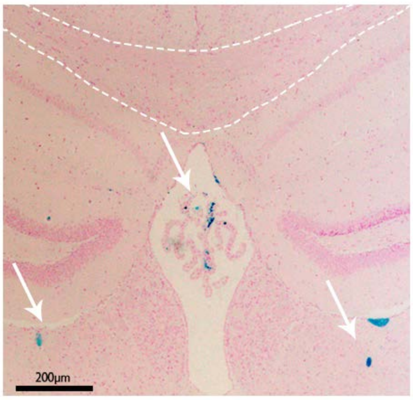

Fig. S1 Prussian blue staining of brain tissue after iron-dextran injection. After injection of iron-dextran in the positive control group, abnormal blue punctate deposits were found in the cerebral tissues of the choroid plexus and the paraventricular brain tissues by PB staining (white arrows), however, no obvious iron deposits were found in the corpus callosum (white dashed frame).

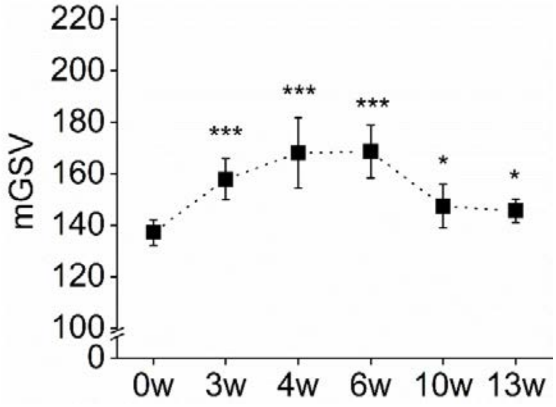

Fig. S2 Trends and comparisons of mGSV of time points of mice model. The mGSV was increased during the demyelination process, reached the peak at the 6w group, and followed by decreased during the remyelination process. Compared with the 0w group, statistically differences are $^{*}p < 0.05$, $^{**}p < 0.01$, $^{***}p < 0.001$.

Table S1 ROI volume independently delineated by two physicians and the intraclass correlation coefficient (ICC) of ROIs.

	n	T_2 (sCC)	T_2 (V3)	b0 (sCC)	filter phase (sCC)
V1 (mm^3)	58	1.116 ± 0.030	0.309 ± 0.001	0.800 ± 0.007	1.092 ± 0.017
V2 (mm^3)	58	1.092 ± 0.029	0.310 ± 0.002	0.798 ± 0.006	1.101 ± 0.017
ICC	—	0.886	0.808	0.864	0.871
P	—	$< 0.001^{***}$	$< 0.001^{***}$	$< 0.001^{***}$	$< 0.001^{***}$

Note: The volume of ROI (V1 and V2) drawn independently by two physicians, using a two-way random-effects model with absolute agreement definition. T_2 (sCC) for the ROI of sCC in T_2WI, and so on. Statistically differences are $^{*}p < 0.05$, $^{**}p < 0.01$, $^{***}p < 0.001$.

Table S2 Multiple post hoc comparisons of MR metrics.

		P value					
		0w	3w	4w	6w	10w	13w
T_2-nor value	0w	—	1.000	$< 0.001^{***}$	$< 0.001^{***}$	$< 0.001^{***}$	0.001^{**}
	3w	1.000	—	$< 0.001^{***}$	$< 0.001^{***}$	0.026^{*}	0.172
	4w	$< 0.001^{***}$	$< 0.001^{***}$	—	1.000	0.001^{**}	0.001^{**}
	6w	$< 0.001^{***}$	$< 0.001^{***}$	1.000	—	$< 0.001^{***}$	$< 0.001^{***}$
	10w	$< 0.001^{***}$	0.026^{*}	0.001^{**}	$< 0.001^{***}$	—	0.570
	13w	0.001^{**}	0.172	0.001^{**}	$< 0.001^{***}$	0.570	—
FA value	0w	—	1.000	0.002^{**}	0.002^{**}	0.002^{**}	1.000
	3w	1.000	—	0.234	0.228	0.215	0.015^{*}
	4w	0.002^{**}	0.234	—	1.000	1.000	$< 0.001^{***}$
	6w	0.002^{**}	0.228	1.000	—	1.000	$< 0.001^{***}$
	10w	0.002^{**}	0.215	1.000	1.000	—	$< 0.001^{***}$
	13w	1.000	0.015^{*}	$< 0.001^{***}$	$< 0.001^{***}$	$< 0.001^{***}$	—
MD value	0w	—	1.000	0.226	$< 0.001^{***}$	$< 0.001^{***}$	1.000
	3w	1.000	—	0.199	$< 0.001^{***}$	$< 0.001^{***}$	1.000
	4w	0.226	0.199	—	0.479	0.008^{**}	0.431
	6w	$< 0.001^{***}$	$< 0.001^{***}$	0.479	—	1.000	$< 0.001^{***}$
	10w	$< 0.001^{***}$	$< 0.001^{***}$	0.008^{**}	1.000	—	$< 0.001^{***}$
	13w	1.000	1.000	0.431	$< 0.001^{***}$	$< 0.001^{***}$	—
RD value	0w	—	1.000	0.029^{*}	$< 0.001^{***}$	$< 0.001^{***}$	1.000
	3w	1.000	—	0.102	$< 0.001^{***}$	$< 0.001^{***}$	1.000
	4w	0.029^{*}	0.102	—	0.838	0.040^{*}	0.005^{**}
	6w	$< 0.001^{***}$	$< 0.001^{***}$	0.838	—	1.000	$< 0.001^{***}$
	10w	$< 0.001^{***}$	$< 0.001^{***}$	0.040^{*}	1.000	—	$< 0.001^{***}$
	13w	1.000	1.000	0.005^{**}	$< 0.001^{***}$	$< 0.001^{***}$	—
AD value	0w	—	1.000	1.000	0.324	0.006^{**}	1.000
	3w	1.000	—	1.000	0.037^{*}	$< 0.001^{***}$	0.426
	4w	1.000	1.000	—	1.000	0.003^{**}	1.000
	6w	0.324	0.037^{*}	1.000	—	1.000	1.000
	10w	0.006^{**}	$< 0.001^{***}$	0.003^{**}	1.000	—	0.283
	13w	1.000	0.426	1.000	1.000	0.283	—

Note: There were many interesting results when comparing those metrics in

pairwise time points. For example, there was a significant difference between the 3w group and the 4w group in the T_2-nor value, but none in the DTI metrics. And there was no significant difference between the 4w group and the 6w group in all metrics. And when comparing the 6w group and the 10w group, the T_2-nor value showed a significant difference for the disease gradually relieved during the remyelination process, but none of DTI metrics. However, there were significant differences between the 10w group and the 13w group in the FA, MD, and RD value; and so on. Combined with Fig. 5 and Table S3, those may suggest that the degree of demyelination and remyelination is different in different CPZ-induced weeks, and to a certain extent, moreover, T_2WI may be more sensitive than FA, RD, and MD. The two sets of p values which symmetrical along the diagonal are the same. For the multiple post hoc comparisons among groups, statistically differences are $^{*}p < 0.05$, $^{**}p < 0.01$, $^{***}p < 0.001$.

Table S3 Post hoc comparisons of mGSV.

		P value					
		0w	3w	4w	6w	10w	13w
mGSV	0w	—	$< 0.001^{***}$	$< 0.001^{***}$	$< 0.001^{***}$	0.034^{*}	0.013^{*}
	3w	$< 0.001^{***}$	—	0.535	0.212	0.090	0.011^{*}
	4w	$< 0.001^{***}$	0.535	—	1.000	0.013^{*}	0.006^{**}
	6w	$< 0.001^{***}$	0.212	1.000	—	0.001^{**}	$< 0.001^{***}$
	10w	0.034^{*}	0.090	0.013^{*}	0.001^{**}	—	1.000
	13w	0.013^{*}	0.011^{*}	0.006^{**}	$< 0.001^{***}$	1.000	—

Note: The results of the multiple comparisons between these groups together showed that the degree of demyelination increased during the demyelination process, and the disease gradually relieved during the remyelination process. The two sets of p values which symmetrical along the diagonal are the same. For the multiple post hoc comparisons among groups, statistically differences are $^{*}p < 0.05$, $^{**}p < 0.01$, $^{***}p < 0.001$.

Table S4 Post hoc statistic effect size and power of each metric.

	Partial η^2	α	1-β
T_2-nor	0.842	0.05	0.9998309
Φ	0.142^{a}	0.05	0.6332481
FA	0.557	0.05	0.9941779
MD	0.570	0.05	0.9945576
RD	0.613	0.05	0.9969195
AD	0.345	0.05	0.9221507
mGSV	0.663	0.05	0.9984217

Note: ^{a}, for the current effect size of SWI (0.142), to achieve power for 90%, the sample size may require 108 mice in total.
